# Supplementary figures and images for: Trypanosoma brucei triggers a broad immune response in the adipose tissue
Source: PLoS Pathog. 2021 Sep 15;17(9):e1009933. doi: 10.1371/journal.ppat.1009933 (PMC8476018; doi:10.1371/journal.ppat.1009933)

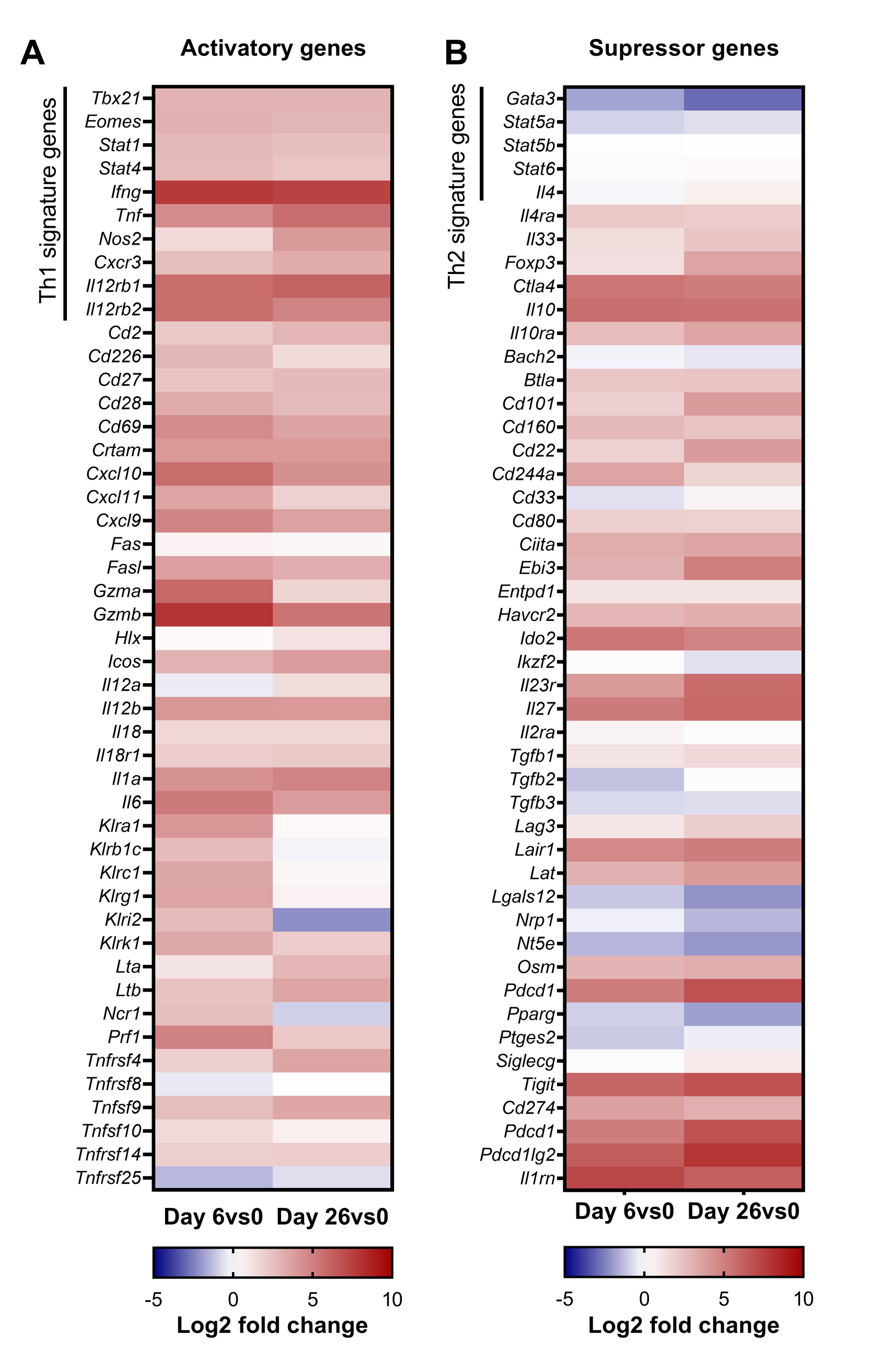

Supplement: S1 Fig — Heat map of the differential expression of genes associated with immune response (A) activation and (B) suppression at days 6 and 26 post-infection relative to non-infected. Gene expression change in Log2 units is denoted in red for up-regulation and in blue for down-regulation. (TIF) [file ppat.1009933.s001.tif]

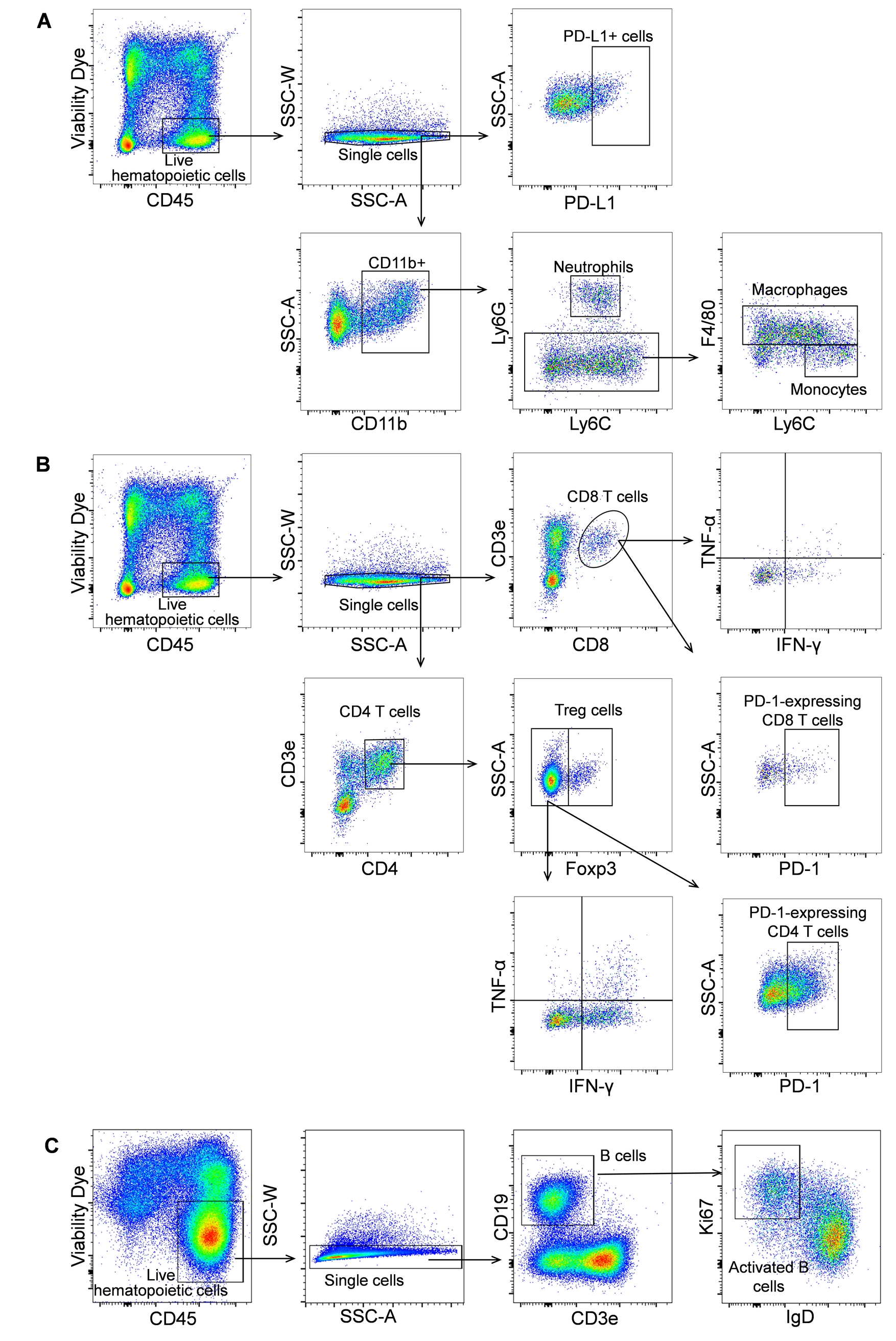

Supplement: S2 Fig — (A) Myeloid gating strategy: live immune cells were gated based on positive expression of CD45 and absence of viability dye signal. Single cells were then identified using SSC-W vs FSC-A gating. Total myeloid cells were selected based on CD11b expression and then subdivided into neutrophils and other myeloid cells based on Ly6G gating. Within the remaining myeloid cells, macrophages and monocytes were identified using F4/80 vs Ly6C gating. (B) Lymphoid gating strategy: single live immune cells were identified as described above and then T cells were identified based on the co-expression of CD3 and CD4 or CD3 and CD8. CD4+ T cells were further subdivided into conventional CD4+ T cells or regulatory T cells based on FoxP3 expression. Effector T cells were identified by gating single or dual expression of TNF-α and IFN-γ within CD3+CD4+FoxP3- or CD3+CD8+ cells (C) B cell gating strategy: Live immune cells were identified as before and then B cells were identified based on expression of CD19 and lack of CD3 expression. Activated B cells were identified based on positive Ki67 expression and absence of IgD expression. (TIF) [file ppat.1009933.s002.tif]

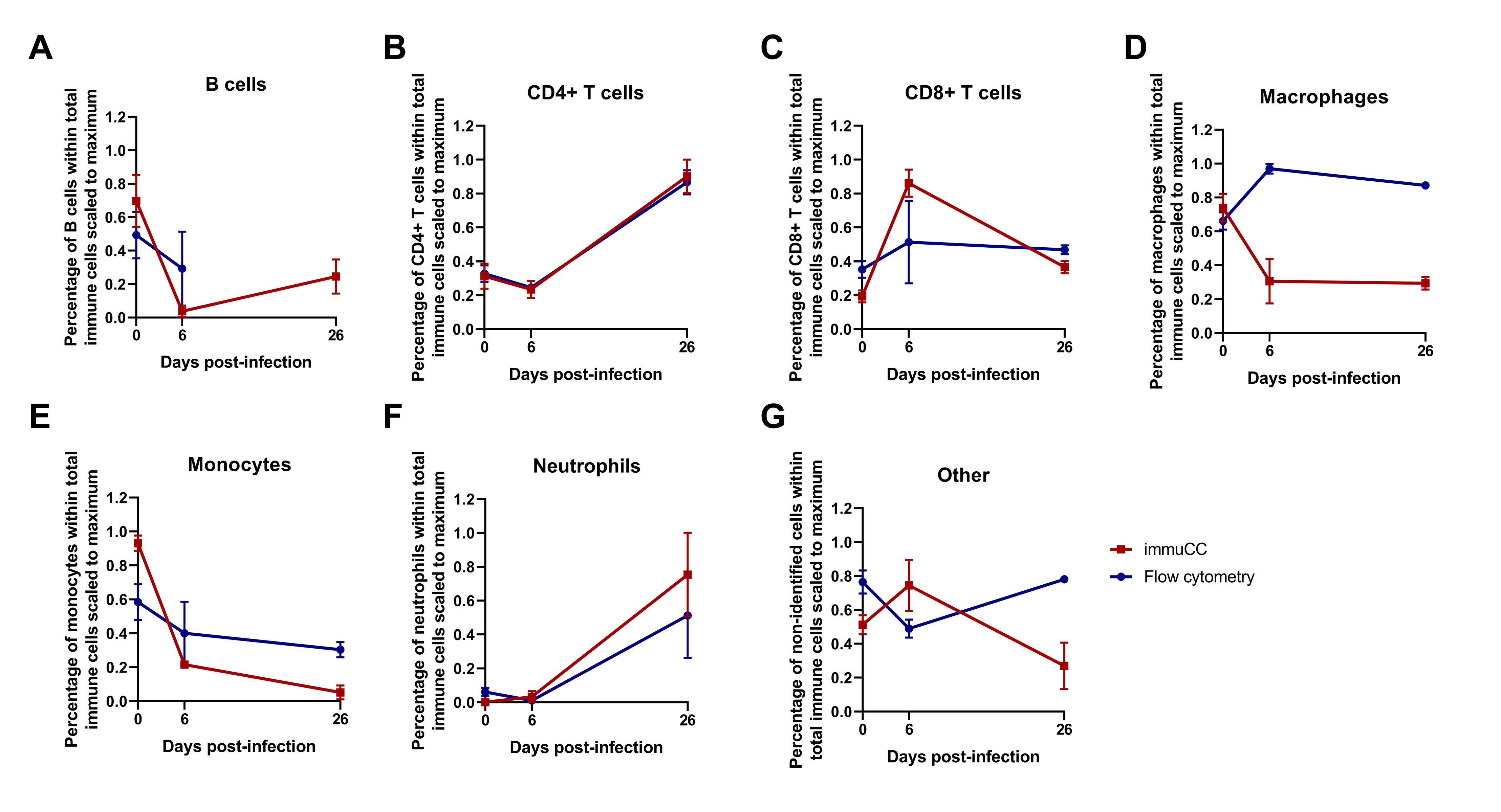

Supplement: S3 Fig — Variation of immune cell subsets between days 0, 6 and 26 post-infection. Data are scaled between 0 and 1, where 1 corresponds to the highest percentual value within each group. (A) B cells, (B) CD4+ T cells, (C) CD8+ T cells, (D) Macrophages, (E) monocytes, (F) neutrophils and (G) other immune cells. (TIF) [file ppat.1009933.s003.tif]

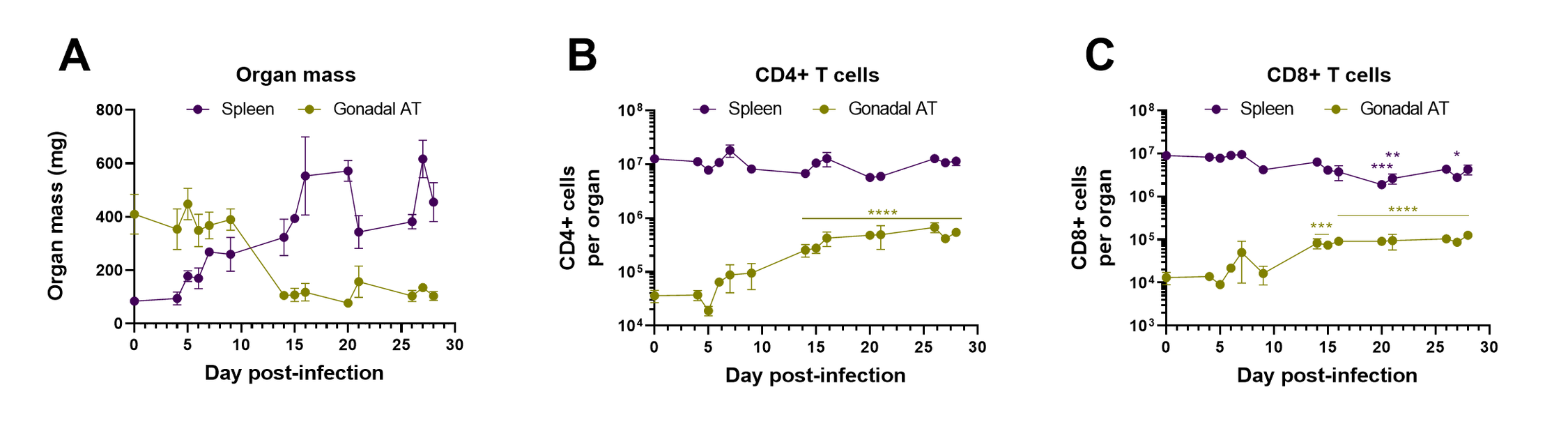

Supplement: S4 Fig — (A) Spleen and gonadal AT mass. (B) CD4+ T cells. (C) CD8+ T cells. Error bars represent the standard error of the mean (n = 2–6 mice per group). Statistical analysis was performed with a two-way ANOVA using Sidak’s test for multiple comparisons. * refers to statistical differences between the group in each time-point and the non-infected group. *, P<0.05; **, P<0.01; ***, P<0.001; ****, P<0.0001. (TIF) [file ppat.1009933.s004.tif]

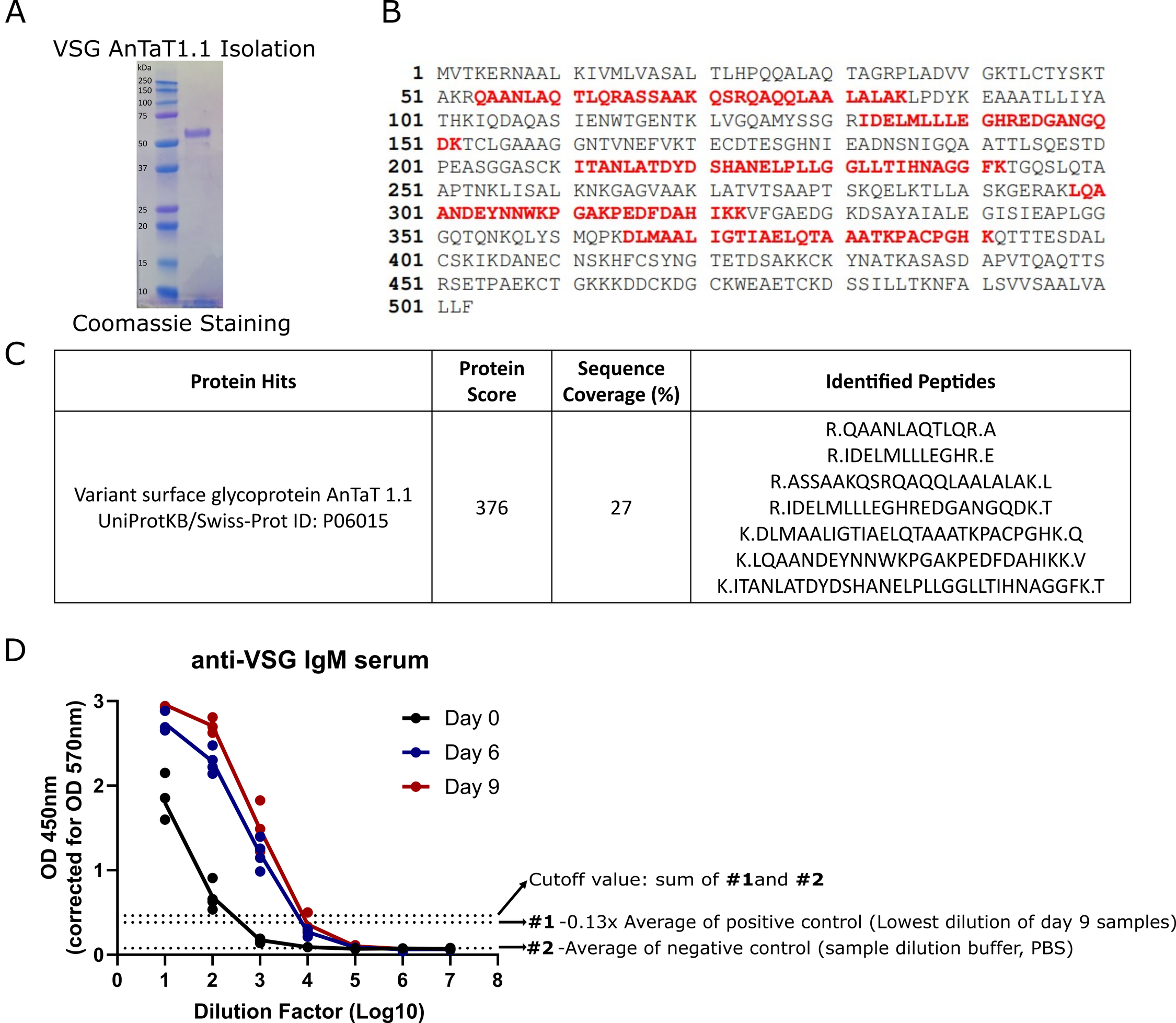

Supplement: S5 Fig — (A) Coomassie stained SDS-PAGE of purified soluble VSG solution denoting a single preeminent band within the predicted size range of VSG. (B) Best peptide match within the SwissProt database (sp|P06015|VSA1_TRYBB, Variant surface glycoprotein AnTaT 1.1 OS = Trypanosoma brucei brucei OX = 5702 PE = 2 SV = 1) with matching peptides depicted in bold red. (C) Summary of protein identification. Protein score is -10*Log(P), where P is the probability that the observed match is a random event. Protein scores greater than 70 are significant (p<0.05). Protein scores are derived from ions scores as a non-probabilistic basis for ranking protein hits. (D) Measurement of anti-VSG IgM titers by ELISA depicting optical density (OD) curves and antibody titer cut off determination. (TIF) [file ppat.1009933.s005.tif]

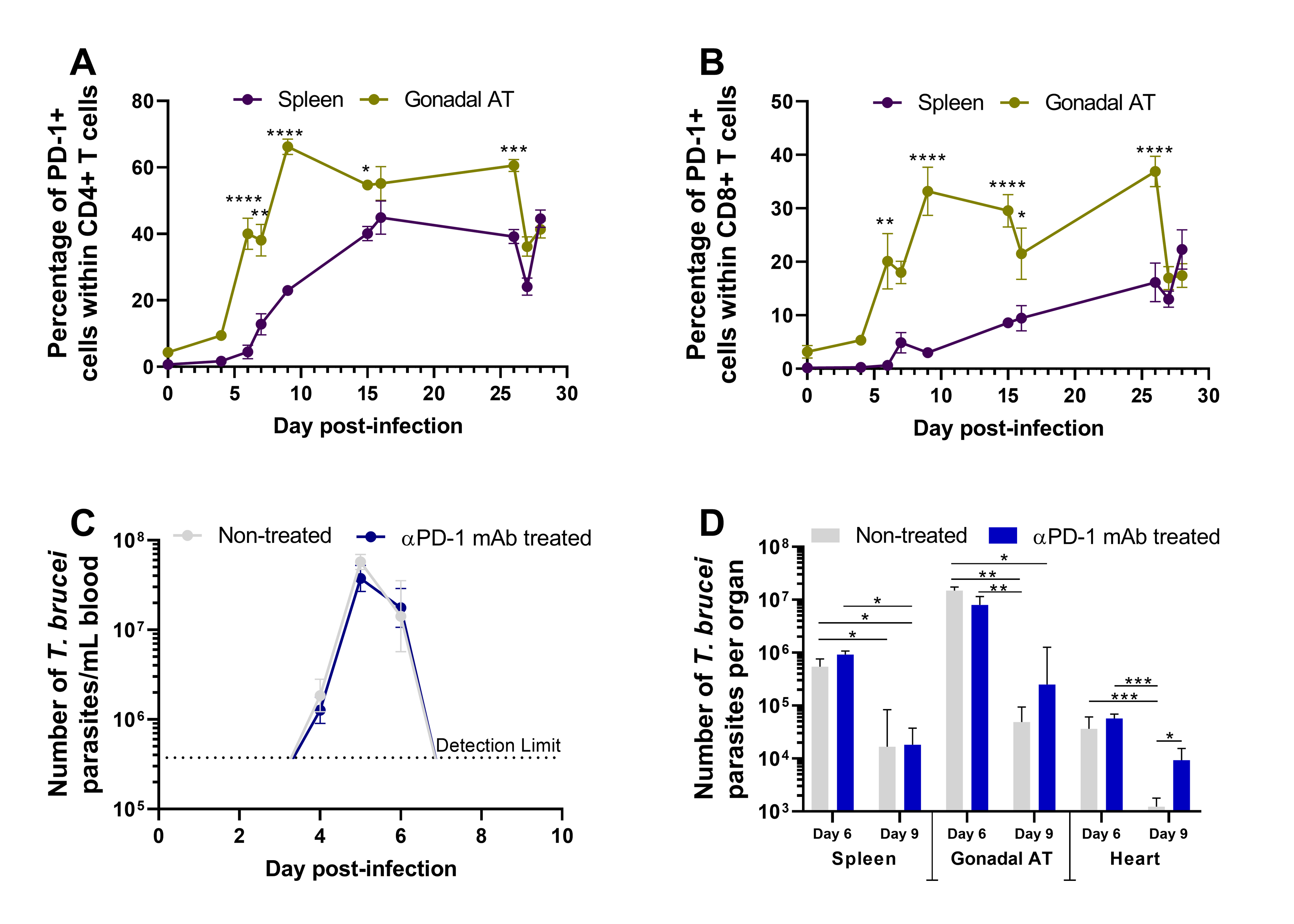

Supplement: S6 Fig — Percentage of PD1+ (A) CD4+ T cells and (B) CD8+ T cells. Effect of anti-PD-1 treatment on (C) parasitemia of mice infected with T. brucei, quantified in a hemocytometer and (D) number of T. brucei parasites quantified by qPCR in spleen, gonadal AT and heart, 6 and 9 days post-infection. Error bars represent the SEM (n = 5 mice per group). Statistical analysis was performed with a two-way ANOVA using Sidak’s test for multiple comparisons. (A-B) * refers to statistical differences between groups. *, P<0.05; **, P<0.01; ***, P<0.001; ****, P<0.0001. (TIF) [file ppat.1009933.s006.tif]

A

|                    | Organ Weight (mg) |     |     |    |     |    |            |     |     |     |     |     |
|--------------------|-------------------|-----|-----|----|-----|----|------------|-----|-----|-----|-----|-----|
| Day post-infection | Spleen            |     |     |    |     |    | gonadal AT |     |     |     |     |     |
| 0                  | 76                | 72  | 98  | 68 | 110 | 82 | 423        | 338 | 374 | 549 | 376 | 398 |
| 4                  | 72                | 68  |     |    |     |    | 398        | 265 |     |     |     |     |
| 5                  | 215               | 173 | 144 |    |     |    | 460        | 499 | 383 |     |     |     |
| 6                  | 233               | 177 |     |    |     |    | 288        | 410 |     |     |     |     |
| 7                  | 266               | 271 |     |    |     |    | 403        | 332 |     |     |     |     |
| 9                  | 323               | 196 |     |    |     |    | 418        | 361 |     |     |     |     |
| 14                 | 398               | 186 | 384 |    |     |    | 116        | 104 | 97  |     |     |     |
| 15                 | 375               | 399 | 406 |    |     |    | 93         | 93  | 136 |     |     |     |
| 16                 | 433               | 383 | 843 |    |     |    | 155        | 95  | 102 |     |     |     |
| 20                 | 615               | 606 | 493 |    |     |    | 79         | 74  | 78  |     |     |     |
| 21                 | 491               | 318 | 368 |    |     |    | 78         | 162 | 168 |     |     |     |
| 26                 | 336               | 429 | 379 |    |     |    | 86         | 127 | 97  |     |     |     |
| 27                 | 745               | 601 | 503 |    |     |    | 146        | 118 | 141 |     |     |     |
| 28                 | 504               | 312 | 549 |    |     |    | 89         | 100 | 122 |     |     |     |

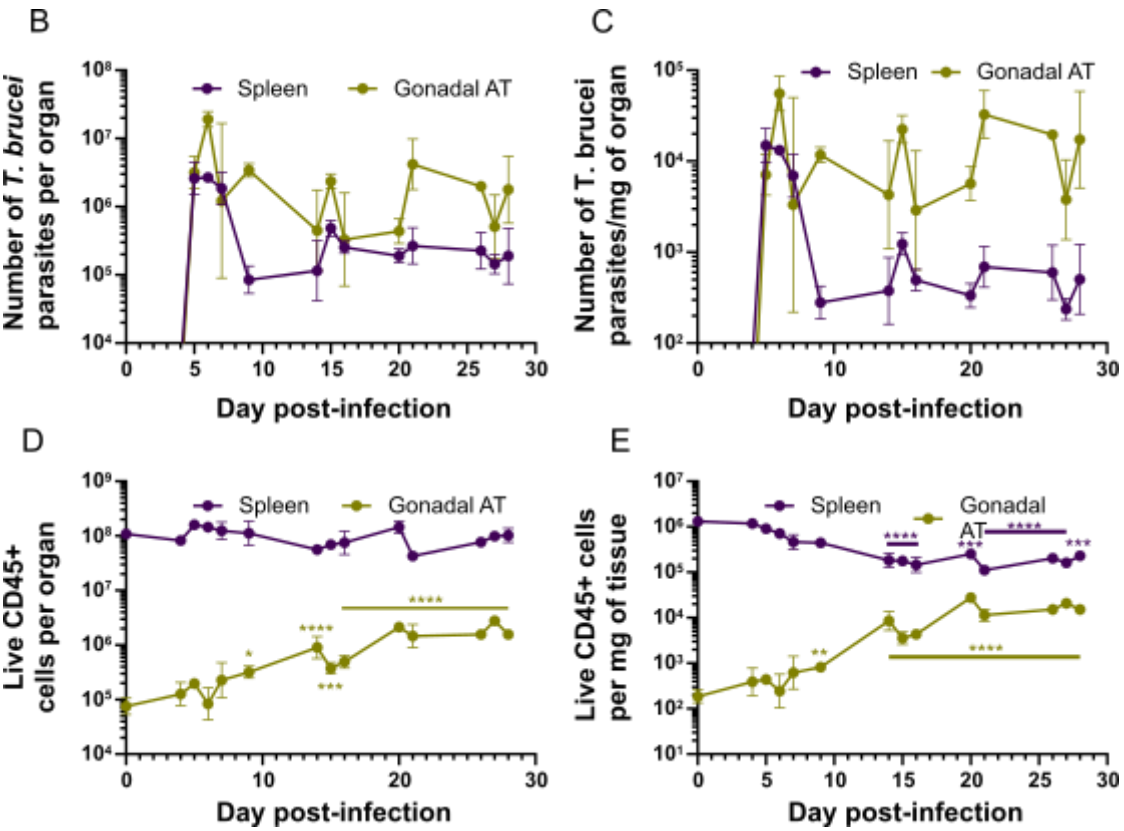

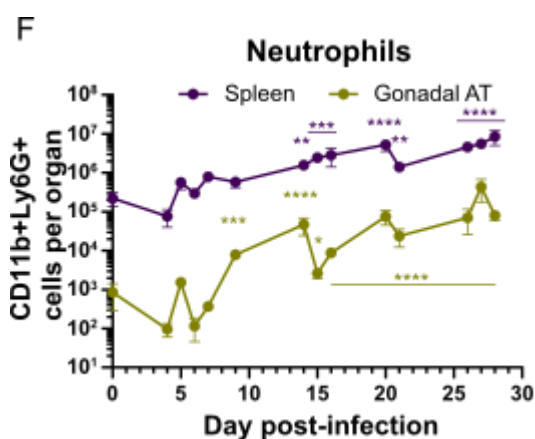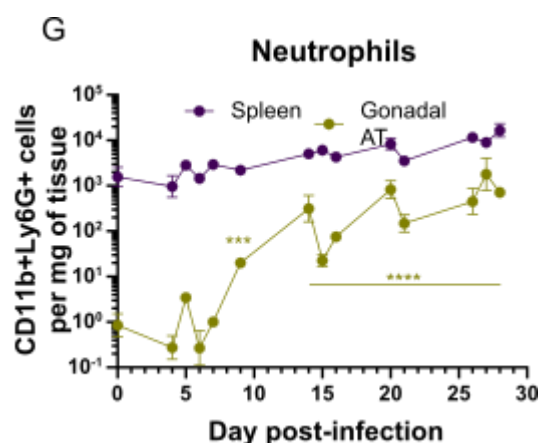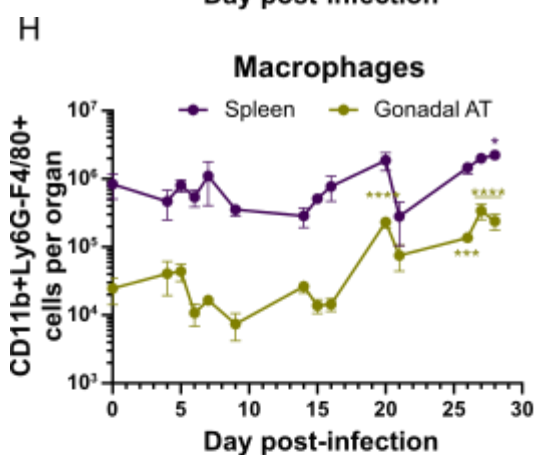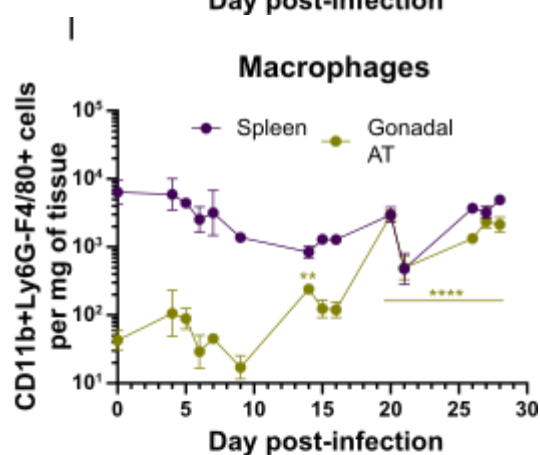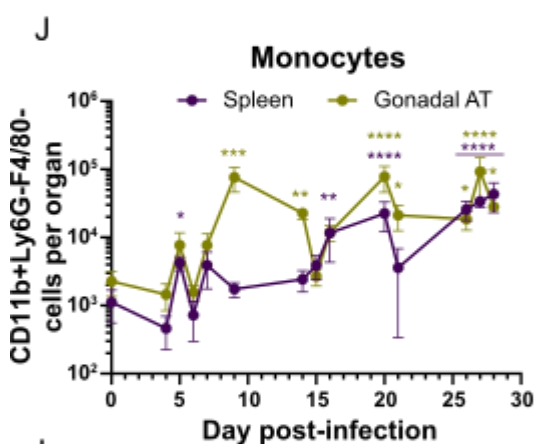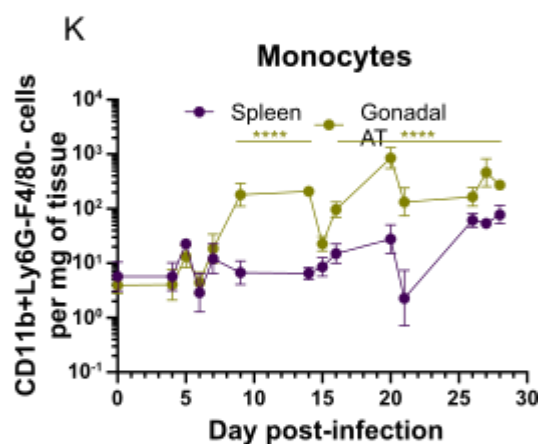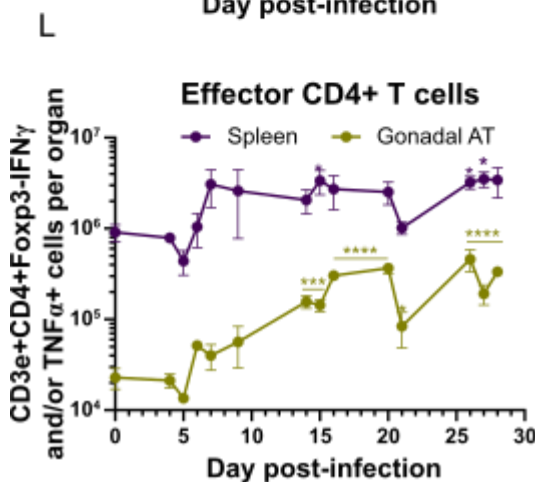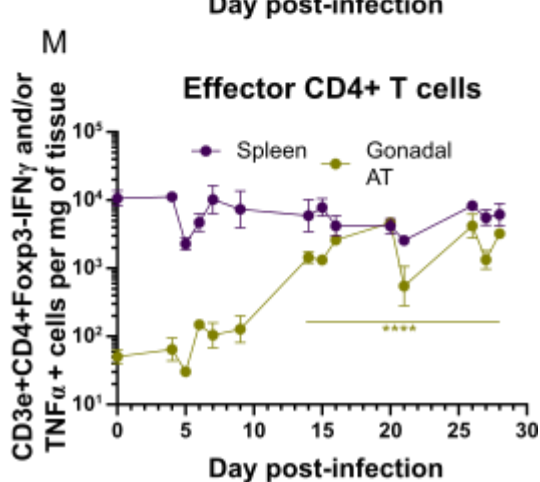

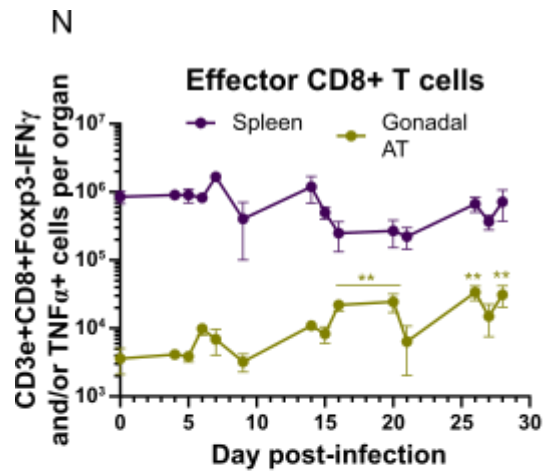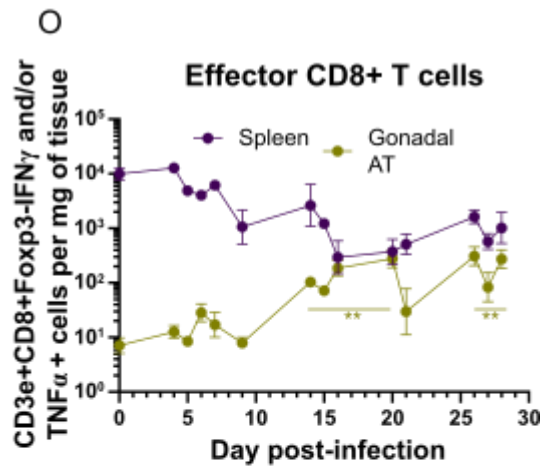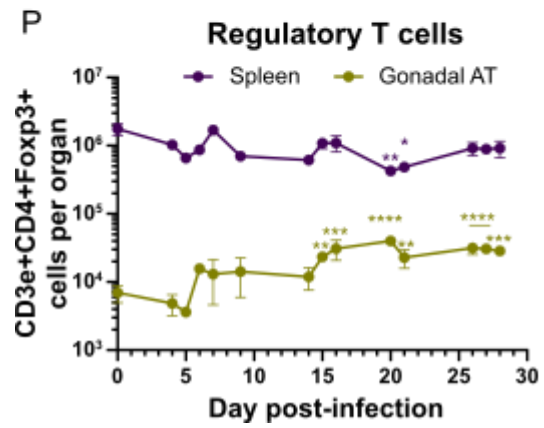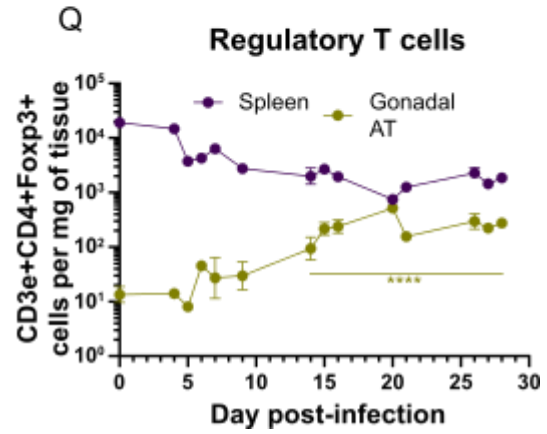

Supplement: S3 File — (A) Weight of spleens and ATs analysed. Data for entire organs and normalized to tissue weight (B-C) parasite burden, (D-E) CD45+ cells, (F-G) neutrophils, (H-I) macrophages, (J-K) monocytes, (L-M) effector CD4+ T cells, (N-O) effector CD8+ T cells and (P-Q) regulatory T cells. (PDF) [file ppat.1009933.s010.pdf]

A

| Day post-infection | Organ Weight (mg) |     |     |     |     |     |     |            |     |     |     |     |     |     |
|--------------------|-------------------|-----|-----|-----|-----|-----|-----|------------|-----|-----|-----|-----|-----|-----|
|                    | Spleen            |     |     |     |     |     |     | gonadal AT |     |     |     |     |     |     |
|                    | 60                | 70  | 60  | 70  |     |     |     | 354        | 366 | 400 | 426 |     |     |     |
| 0                  | 254               | 247 | 323 | 382 | 258 | 311 | 320 | 572        | 581 | 242 | 329 | 364 | 428 | 301 |
| 6                  | 450               | 325 | 110 | 413 | 425 | 362 | 336 | 183        | 250 | 271 | 125 | 180 | 220 | 194 |
| 9                  |                   |     |     |     |     |     |     |            |     |     |     |     |     |     |

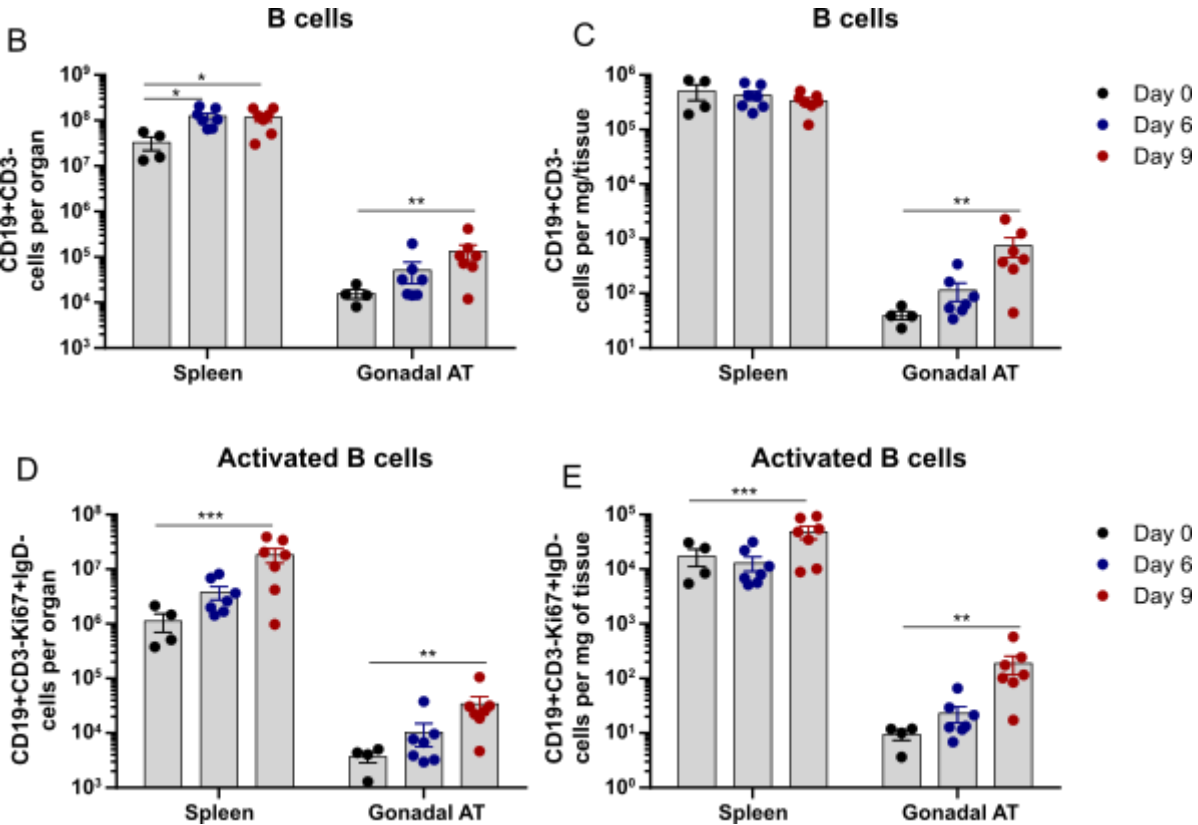

Supplement: S4 File — (A) Weight of spleens and ATs analysed. Data for entire organs and normalized to tissue weight (B-C) B cells and (D-E) activated B cells. (PDF) [file ppat.1009933.s011.pdf]
