# Supplementary material for: Trypanosoma brucei triggers a broad immune response in the adipose tissue
Source: PLoS Pathog. 2021 Sep 15;17(9):e1009933. doi: 10.1371/journal.ppat.1009933 (PMC8476018; doi:10.1371/journal.ppat.1009933)
Supplement: S1 Table — (DOCX) [file ppat.1009933.s007.docx]

**S1 Table**. Mapping information of RNA-Seq reads in samples from infected AT.

| **Sample** | **Total # reads** | **# reads mapped to T. brucei** | **% *T. brucei* reads** | **# reads mapped to mouse** | **% mouse reads** |
| --- | --- | --- | --- | --- | --- |
| D0.1 | 46435679 | 361377 | 0.8 | 44507134 | 95.8 |
| D0.2 | 37539439 | 301273 | 0.8 | 36140468 | 96.3 |
| D0.3 | 43654064 | 329134 | 0.8 | 41916511 | 96.0 |
| D6.1 | 45755070 | 3662129 | 8.0 | 39651842 | 86.7 |
| D6.2 | 36562366 | 3523271 | 9.6 | 31433268 | 86.0 |
| D6.3 | 37547584 | 690724 | 1.8 | 35552484 | 94.7 |
| D26.1 | 50699601 | 2193477 | 4.3 | 46274150 | 91.3 |
| D26.2 | 54368447 | 3068465 | 5.6 | 48765774 | 89.7 |
