# Supplementary material for: Trypanosoma brucei triggers a broad immune response in the adipose tissue
Source: PLoS Pathog. 2021 Sep 15;17(9):e1009933. doi: 10.1371/journal.ppat.1009933 (PMC8476018; doi:10.1371/journal.ppat.1009933)
Supplement: S5 File — Weight of organs used from infected (A) Rag2-/-, (B) Jht-/-, (C) Ifng-/-, (D) C3-/- and (E) anti-PD1 treated mice, with respective controls. Data for parasite numbers in the entire organ or normalized to tissue weight for infected (F-G) Rag2-/-, (H-I) Jht-/-, (J-K) Ifng-/-, (L-M) C3-/- and (N-O) anti-PD1 treated mice, with respective controls. (PDF) [file ppat.1009933.s012.pdf]

A

	**(*Rag2-/-*)** Tissue Weight (mg)																	
Day post-infection	Spleen						AT						Heart					
6 WT	198	287	251	280			310	295	423	317			97	102	104	114		
6 *Rag2-/-*	69	71	127	53			188	240	239	291			87	101	119	104		
9 WT	282	401	190	324			239	194	261	313			102	116	116	117		
9 *Rag2-/-*	212	228	198	197	219	219	142	120	162	175	200	131	95	90	87	108	93	87

B

	**(*Jht-/-*)** Tissue Weight (mg)																				
Day post-infection	Spleen							AT							Heart						
6 WT	254	247	323	381	258	310	320	572	581	243	329	364	429	302	145	133	149	143	151	133	181
6 *Jht-/-*	128	119	222	221	135	122	126	482	437	420	377	375	365	366	136	130	128	167	122	100	105
9 WT	450	325	110	413	363	337		183	249	271	125	180	220	194	176	116	141	137	139	100	111
9 *Jht-/-*	164	179	208	171	225	195	213	317	341	365	227	135	232	199	131	107	119	125	195	91	98

C

	(Ifng-/-) Tissue Weight (mg)											
Day post-infection	Spleen				AT				Heart			
6 WT	301	255	301	239	318	329	314	408	124	99	127	116
6 *Ifng-/-*	359	276	335	305	260	266	246	246	113	102	117	95
9 WT		438	400	422	246	292	270	271	109	109	111	103
9 *Ifng-/-*	307	266	353	291	215	161	229	208	98	92	91	105

D

	**(*C3-/-*)** Tissue Weight (mg)														
Day post-infection	Spleen					AT					Heart				
6 WT	222	237	242	186		356	346	250	302		120	98	110	107	
6 *C3-/-*	317	368	360	265		162	226	184	290		120	114	132	137	
9 WT	237	405	373	337		246	306	182	214		101	124	104	110	
9 *C3-/-*	457	354	502	306	441	188	348	236	196	314	115	112	117	109	110

E

	**(anti-PD-1)** Organ Weights (mg) (WT shared with IFN-/- experiment)											
Day post-infection	Spleen				AT				Heart			
6 WT	301	255	301	239	318	329	314	408	124	99	127	116
6 anti-PD1	233	310	193	276	352	320	383	386	110	102	117	115
9 WT		438	400	422	246	292	270	271	109	109	111	103
9 anti-PD1	289	357	357	395	328	183	269	216	91	103	112	130

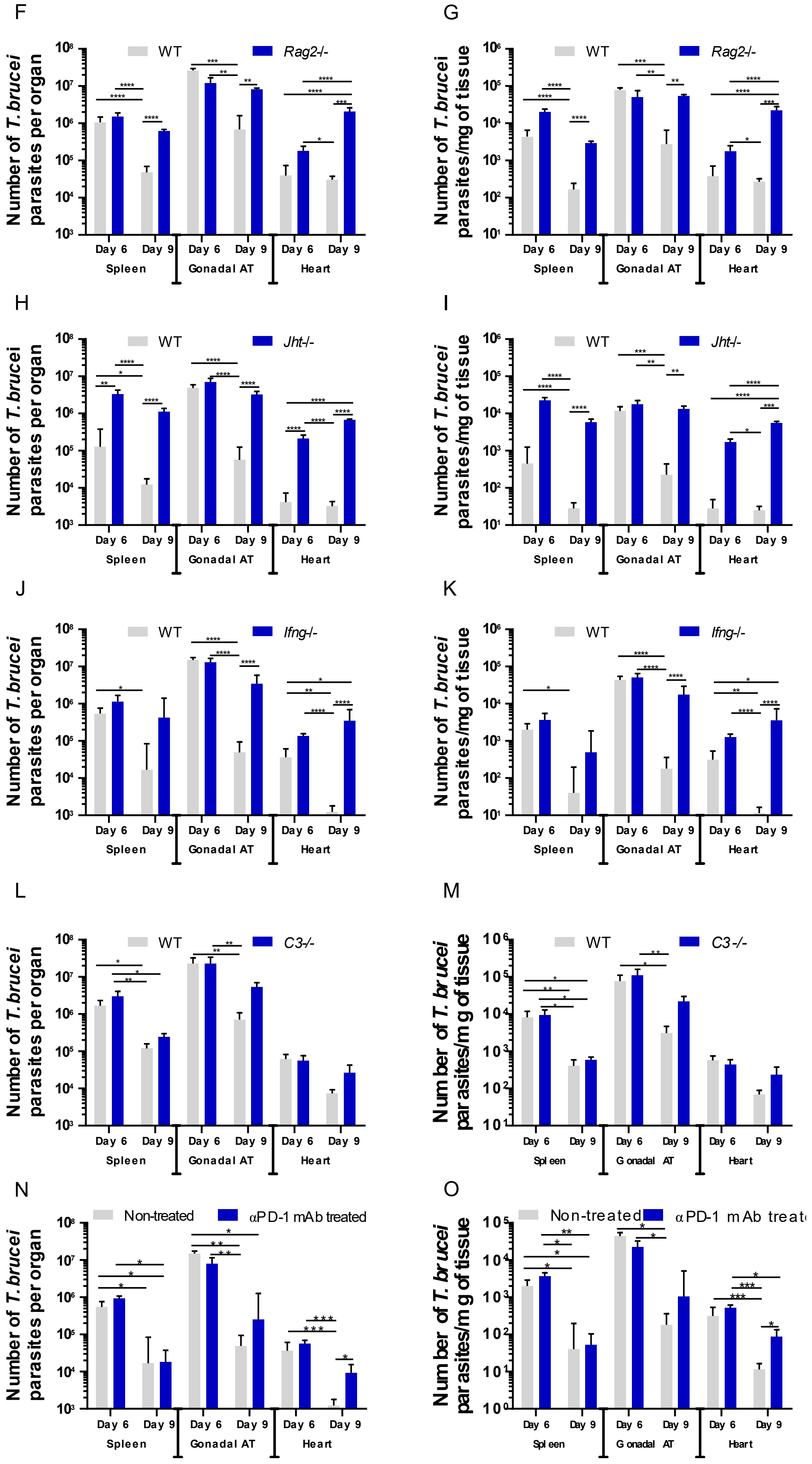
F
G
H
I
J
K
L
M
N
O
Number of T. brucei parasites per organ
Number of T. brucei parasites/mg of tissue
WT
Rag2-/-
Jht-/-
Ifng-/-
C3-/-
Non-treated
αPD-1 mAb treated
Day 6
Day 9
Spleen
Gonadal AT
Heart
