# Supplementary material for: Trypanosoma brucei triggers a broad immune response in the adipose tissue
Source: PLoS Pathog. 2021 Sep 15;17(9):e1009933. doi: 10.1371/journal.ppat.1009933 (PMC8476018; doi:10.1371/journal.ppat.1009933)
Supplement: Attachment — Submitted filename: Machado_Editing.docx [file ppat.1009933.s013.docx]

1) We note that the following authors have not yet confirmed their authorship on this manuscript: Karine Serre 
I believe Karine Serre has now confirmed her authorship.

2) We do not publish any copyright or trademark symbols that usually accompany proprietary names, eg (R), (C), or TM  (e.g. next to drug or reagent names). Therefore please remove all instances of trademark/copyright symbols throughout the text, including Pierce™ on page 34, Nunc™  MaxiSorp™ on page 35.
The symbols have been removed from the “clean” manuscript and the one with “tracked changes”.

3) Please ensure that the funders and grant numbers match between the Financial Disclosure field and the Funding Information tab in your submission form. Note that the funders must be provided in the same order in both places as well. Currently, the order of the funders and grants differs in both places.
I think our manuscript was missing a Financial Disclosure field altogether. We have now included it.

4) Please amend your detailed Financial Disclosure statement. This is published with the article, therefore should be completed in full sentences and contain the exact wording you wish to be published.
i). State what role the funders took in the study. If the funders had no role in your study, please state: “The funders had no role in study design, data collection and analysis, decision to publish, or preparation of the manuscript.”
ii). If any authors received a salary from any of your funders, please state which authors and which funders.
We have now included a financial disclosure statement in the manuscript which says:

"This work was supported by the European Research Council (FatTryp, ref. 771714) awarded to LMF, by Fundação para a Ciência e Tecnologia (CEECIND/03322/2108) awarded to LMF, (CEECIND/00697/2018) awarded to KS, (PD/BD/128286/2017) awarded to HM, (SFRH/BPD/89833/2012) awarded to ST, (IMM/BI/83-2017 through PTDC/BIM-MET/4471/2014) awarded to TB-R and by the National Institutes of Health (NIGMS K99GM132557) awarded to FR-F. The funders had no role in study design, data collection and analysis, decision to publish, or preparation of the manuscript."

5) When completing the data availability statement of the submission form, you indicated that  "RNA-Seq data is publicly available in the ArrayExpress database under the accession number E-MTAB-4061 and E-MTAB-7596.". However, you also checked the following box: “Tick here if the URLs/accession numbers/DOIs will be available only after acceptance of the manuscript for publication so that we can ensure their inclusion before publication.” If this box was checked in error, and there is no additional data to deposit, please ensure this box is unticked when you resubmit.

We included a new section at the end of Methods called “Data Availability”:

RNA-Seq data is publicly available in the ArrayExpress database under the accession number E-MTAB-4061 and E-MTAB-7596.
